# Characteristics of GPs responding to an educational intervention to minimise inappropriate prescriptions: subgroup analyses of the Rx-PAD study

**DOI:** 10.3399/bjgpopen18X101373

**Published:** 2018-04-21

**Authors:** Sture Rognstad, Mette Brekke, Ibrahimu Mdala, Arne Fetveit, Svein Gjelstad, Jørund Straand

**Affiliations:** 1 University Lecturer, General Practice Research Unit (AFE), Department of General Practice, Institute of Health and Society, University of Oslo, Oslo, Norway; 2 Professor, General Practice Research Unit (AFE), Department of General Practice, Institute of Health and Society, University of Oslo, Oslo, Norway; 3 Statistician, General Practice Research Unit (AFE), Department of General Practice, Institute of Health and Society, University of Oslo, Oslo, Norway; 4 Assistant Professor, General Practice Research Unit (AFE), Department of General Practice, Institute of Health and Society, University of Oslo, Oslo, Norway; 5 Assistant Professor, General Practice Research Unit (AFE), Department of General Practice, Institute of Health and Society, University of Oslo, Oslo, Norway; 6 Assistant Professor, General Practice Research Unit (AFE), Department of General Practice, Institute of Health and Society, University of Oslo, Oslo, Norway

**Keywords:** Intervention study, feedback, general practice, inappropriate prescribing, aged, randomized controlled trial, peer group

## Abstract

**Background:**

Interventions aimed at improving GPs’ prescribing practice usually apply a 'one size fits all' when analysing intervention effects. Few studies explore intervention effects by variables related to the GPs’ age, sex, specialist status, practice type (single-handed versus group), practice setting (urban versus rural), and baseline performance regarding the target of an intervention.

**Aim:**

To explore the characteristics of the GPs responding to a comprehensive educational intervention.

**Design & setting:**

A secondary analysis of a cluster, randomised educational intervention in Norwegian general practice. Pre-intervention data were captured from January 2005 to December 2005, and post-intervention data from June 2006 to June 2007. The intervention was carried out from January to June 2006.

**Method:**

Eighty continuing medical education (CME) groups, including 449 GPs aged 27–68 years, were randomly allocated to either an education intervention arm (41 groups, 250 GPs) or a control arm (39 groups, 199 GPs). The primary outcome was GPs' change in potentially inappropriate prescriptions (PIPs) per 100 prescriptions issued to patients aged ≥70 years. The interaction between intervention outcome and variables related to the GPs and their practices were tested.

**Results:**

Improvements in prescribing were highest among GPs aged 57–68 years (incidence rate ratio [IRR] = 0.77 [95% confidence interval {CI} = 0.73 to 0.81]), those who were specialists (IRR = 0.80 [95% CI = 0.78 to 0.82]), and those who worked in single-handed practices (IRR = 0.75 [95% CI = 0.68 to 0.83]), among GPs with 2.4 to 2.9 PIPs per 100 prescriptions at baseline (IRR = 0.74 [95% CI = 0.70 to 0.78]), and GPs with ≥15 prescriptions per patient per year at baseline (IRR = 0.77 [95% CI = 0.73 to 0.80]).

**Conclusion:**

The GPs with the lowest adherence to recommended practice at baseline improved their practice most.

## How this fits in

This is the first GP-based intervention study to investigate the influence of GPs’ characteristics on their change of prescribing practice across a broad spectrum of therapeutic areas. Earlier reviews have reported that the relative effectiveness of audit and feedback is likely to be greater when baseline adherence to recommended practice is low. This subgroup analysis of a previous large-scale educational intervention confirms this. GPs in the oldest age group had relatively high levels of potentially inappropriate prescribing at baseline, and thus were in greatest need of improvement. They responded best to the educational intervention.

## Introduction

Older people are generally more vulnerable to adverse drug reactions due to altered pharmacokinetics and pharmacodynamics, comorbidity, and polypharmacy.^1^ Inappropriate medication use in frail older people puts them at increased risk for drug-related harms, which may cause impaired quality of life, increased morbidity, and even death.^2,3^ In general practice, drugs or combinations of drugs considered potentially inappropriate for older people are reported to make up 5–34% of all prescriptions issued to older patients.^4–8^ The *Rx*-PAD study, a large educational intervention study carried out among 449 GPs in Norway, was carried out betwen 2005–2007.^9^ The aim of that intervention was to improve GPs’ prescribing practice for older patients (≥70 years) in terms of reducing the prevalence of a number of listed explicit criteria for PIPs (Box 1). Average outcome figures 1 year after the intervention revealed that the average proportion of PIPs per 100 prescriptions per GP in the intervention group, as compared with the control group, was reduced by 13% in relative terms.^9^ However, it is not known which GPs responded in line with the aims of the intervention. Such information may be relevant for the planning of future comparable educational intervention studies.^10^

**Box 1.** Thirteen explicit criteria for PIPs used for assessing the appropriateness of GPs’ prescriptions to older patients (≥70 years).

**Single drugs**	**Combination of drugs**
1. Tricyclic antidepressants *Amitriptyline* *Doxepine* *Trimipramine*	8. Combination of a beta-blocker with either *Verapamil or* *Diltiazem*
2. First generation antihistamines *Dexchlorpheniramine* *Promethazine* *Alimemazine* *Hydroxycine*	9. Combination of an NSAID and warfarin
3. First generation (low potency) *Chlorpromazine* *Chlorprotixene* *Levoprometazine* *Prochlorperazine*	10. Combination of an NSAID (or a Cox-2 inhibitor) with an ACE-inhibitor (or an ARB)
4. Long-acting benzodiazepines *Carisoprodol*	11. Combination of an NSAID and a SSRI
5. Muscle relaxant *Carisoprodol*	12. Combination of an NSAID and a diuretic
6. Strong analgesics *Propoxyphene* *Pethidine* *Opioids with spasmolytics *	13. Concurrent use of ≥3 psychotropic drugs within classes: *Opioid analgesics* *Hypnotics* *Tranquillisers * *Antipsychotics* *Antidepressants*
7. Theophylline tablets	

ACE = angiotensin converting enzyme. ARB = Angiotensin receptor blocker. COX = cyclooxygenase. NSAID = non-steroidal anti-inflammatory drug. PIP = potentially inappropriate prescription. SSRI = selective serotonin reuptake inhibitor.

The aim of this study was to re-analyse the effect of the *Rx*-PAD intervention study^9 ^by subgroup, analysing the intervention effect by characteristics linked to the GPs or their practices.

## Method

In this subgroup analysis of the *Rx*-PAD study,^9 ^outcome measures of seven explanatory variables were explored. In randomised trials, subgroup analyses are commonly applied to examine how various explanatory variables may be associated with the intervention outcome. Subgroup analyses are not based on randomised comparisons and their limitations are well-established: false positives due to multiple comparisons, false negatives due to inadequate power.^10^ Although it is almost impossible to fully eliminate such faults in a subgroup analysis, the aim of the present study was to formally test the interaction between the intervention and subgroups among the GPs by using a strict *P*-value to control for multiple testing.

The *Rx*-PAD study was designed to assess the effects of an educational intervention given to GPs, which aimed at reducing their prescribing rates of PIPs for older patients.^11^ Eighty CME groups comprising 449 GPs were allocated to either intervention (41 groups, 250 GPs) or control (39 groups, 199 GPs). The control group participated in a corresponding educational intervention to improve the prescribing of antibiotics.^12^ The two groups were control group for each other. All GPs were part of the Norwegian GP patient-list system and the GPs’ prescription data were extracted from the Norwegian Prescription Database (NorPD).^13^

PIPs were defined using the 13 explicit items given in Box 1. The clinical relevance of these was validated by the participating GPs in regional meetings^11^ and in focus group interviews among participating tutors and GPs.^14^ Briefly, the intervention comprised an individual prescription report (sent to all GPs in the intervention group) and two CME-group sessions chaired by a tutor from the research group.^8,9,11^ A prescription report disclosed a GP’s prescribing practice for the 13 criteria at baseline (NorPD-data from the year prior to the intervention) and as compared with average figures for all participants. During the two CME-group sessions, the tutor presented the evidence for the various criteria (Box 1) and facilitated a structured discussion between the GPs. Based on their individual feedback reports, this discussion aimed to identify individual targets for improvements.^9^ Outcomes were based on NorPD-prescription data from the 1-year period after the intervention, as compared with baseline figures and corresponding figures from the control group.^9^ This study explored the outcome measure (that is, the number of PIPs per 100 prescriptions) for the seven following subgroups: GPs' age, sex, specialty in general practice or not, setting in a single-handed or group practice, urban or rural practice location, GPs’ prevalence of PIPs at baseline relative to prescriptions issued for older patients, and, finally, GPs’ baseline prescribing activity for older patients. The latter defined baseline as annual number of prescriptions per older patient who was issued any prescriptions.

### Statistical methods

The overall and subgroup prevalence of PIPs at pre- and post-intervention were estimated as proportions of all prescriptions at pre- and post-intervention respectively. The prevalence of PIPs at post-intervention was compared to the prevalence of PIPs at pre-intervention using the relative risk (RR) with 95% CIs. An RR of 1.0 indicates identical prevalence of PIPs at pre- and post-intervention. An RR >1.0 indicates an increase of the prevalence of PIPs at post-intervention relative to pre-intervention, while a RR <1.0 indicates a decrease of the prevalence of PIPs at post-intervention compared to pre-intervention.

The Poisson model is usually considered the basic model for modeling counts and rates. In this cluster randomised trial, each CME group defined a cluster. Therefore, data on rates of PIPs defined as the number of PIPs per 100 prescriptions were analysed using the Poisson regression model extended by including CME group random effects to account for data clustering. Estimates of IRRs with 95% CIs were obtained from the Poisson multilevel models and represent the change in PIPs per 100 prescriptions at post-intervention relative to baseline. If the IRR is <1.0, then a reduction in PIPs was observed, and if it is >1.0, then an increase in PIPs was observed. When the IRR = 1.0, then there was no change in PIPs per 100 prescriptions between pre- and post-intervention. Two steps preceded the modeling of PIPs using the Poisson model. The significance level used was *P*<0.01.

First, a non-stratified model was fitted to investigate the rate of change of PIPs in the intervention group versus the control group. Using this model, it was possible to investigate the rate of change of PIPs between pre- and post-intervention within the control and intervention groups. The model was adjusted for the following GP characteristics: age, sex, specialisation, type and setting of the practice, baseline values of mean PIPs per 100 prescriptions, and number of prescriptions per older patient. The GPs’ age, their annual baseline figures for PIP prevalence, and average number of prescriptions per older patient were split into quintiles by numbers of GPs.

Secondly, the changes in prescription rates of PIPs in intervention and control groups by the seven subgroups of GPs’ characteristics were compared. Assuming no difference in intervention effect between the seven independent subgroups, the probability of at least one significant subgroup is given by 1 − (1 − *α*)7 = 0.30 for *α* = 0.05. The overall type I error rate was controlled conservatively by using *α* * = *α* / 7 = 0.007 in each of the seven subgroup analyses. All analyses were performed in StataSE (version 13).

## Results 

Summary data showing the prevalence of PIPs pre- and post-intervention are given in Table 1. After the intervention, there were 19 754 PIPs, which accounted for 1.79% of total 1 104 391 prescriptions issued by 449 GPs to patients aged ≥70 years. The results show a 25% reduction in PIPs after intervention in the intervention group, and a 13% reduction in the control group.

**Table 1.** Unadjusted summary data showing the prevalence of PIPs and estimates of RR with 95% CIs in different subgroups of GP and drug-related characteristics at pre- and post-intervention. The total number of prescriptions pre-intervention was 1 034 033 and in the post-intervention period there were 1 104 391 prescriptions.

	GPs, *n*	PIPs prevalence, %		Relative risk^a^ (95% CI)
		Pre-intervention (*n* = 23 184)	Post-intervention (*n* = 19 754)	
**Overall group**	449	2.24	1.79	0.80 (0.78 to 0.81)
Intervention	250	1.32	0.99	0.75 (0.73 to 0.77)
Control	199	0.92	0.80	0.87 (0.84 to 0.89)
**Sex**				
Female	139	0.44	0.37	0.83 (0.80 to 0.87)
Male	310	1.80	1.42	0.79 (0.77 to 0.81)
**Practice setting**				
Rural	208	1.01	0.80	0.79 (0.77 to 0.81)
Urban	241	1.23	0.99	0.80 (0.78 to 0.82)
**Type of practice**				
Single-handed	38	0.25	0.20	0.80 (0.75 to 0.84)
Group practice	411	1.99	1.59	0.80 (0.78 to 0.81)
**GP specialist**				
No	63	0.20	0.18	0.89 (0.84 to 0.95)
Yes	386	2.04	1.61	0.79 (0.77 to 0.80)
**Age group, years^b^**				
27–42	84	0.23	0.22	0.95 (0.90 to 1.01)
43–48	102	0.42	0.32	0.77 (0.74 to 0.81)
49–52	75	0.38	0.29	0.77 (0.73 to 0.80)
53–56	98	0.55	0.44	0.79 (0.77 to 0.83)
57–68	90	0.66	0.51	0.78 (0.75 to 0.80)
**Mean PIPs per 100 prescriptions** **1-year pre-intervention^b^**				
0.5–1.6	89	0.24	0.22	0.91 (0.86 to 0.96)
1.7–2.0	90	0.39	0.33	0.85 (0.81 to 0.89)
2.1 –2.3	90	0.49	0.38	0.77 (0.74 to 0.80)
2.4–2.9	90	0.57	0.44	0.77 (0.74 to 0.80)
3.0–6.3	90	0.55	0.43	0.77 (0.74 to 0.80)
**Prescriptions per patient in ** **the 1 year pre-intervention** **period^b^**				
1.9–8.6	89	0.21	0.20	0.95 (0.90 to 1.01)
8.7–10.7	90	0.35	0.28	0.79 (0.76 to 0.83)
10.8–12.8	90	0.44	0.35	0.79 (0.76 to 0.83)
12.9–14.9	90	0.56	0.44	0.80 (0.77 to 0.83)
15.0–32.6	90	0.68	0.51	0.76 (0.73 to 0.79)

^a^Relative risk indicates how large the prevalence of PIPs at post-intervention was relative to the prevalence at pre-intervention. Relative risk estimates <1 indicate a reduction of PIPs. ^b^Divided into quintiles by number of GPs. PIP = potentially inappropriate prescription. RR = relative risk.

### Overall effect of intervening, adjusted for GPs characteristics

IRRs from the Poisson multilevel models represent the change in PIPs per 100 prescriptions at post-intervention relative to baseline. In Table 2, the rate of change of PIPs in the intervention group versus the control group is given after adjusting for sub-group characteristics of the GPs. There was no significant difference in PIPs at baseline between the control group and the intervention group, (IRR = 1.10 [95% CI = 0.97 to 1.25], *P* = 0.15). After intervention, the rate of PIPs per 100 prescriptions decreased significantly, by 7% (IRR = 0.93 [95% CI = 0.90 to 0.96]) in the control group and 20% (IRR = 0.80 [95% CI = 0.78 to 0.82]) in the intervention group (*P*<0.01), as shown in Table 2. Compared to GPs aged 27–42 years, the change in PIP rates among GPs in the age groups 43–48 and 49–52 years was significantly lower, by 15% and 8%, respectively. However, a relatively higher rate of PIPs post-intervention was observed among GPs in the age group 57–68 years, compared to GPs aged 27–42 years. The analysis also showed that GPs who had more PIPs per 100 prescriptions during the 1-year pre-intervention period (baseline), had more PIPs per 100 prescriptions post-intervention. The rate of PIPs per 100 prescriptions was 40% higher among male GPs compared to female GPs, and GPs in urban areas had significantly higher PIPs than peers in rural areas (see Table 2).

**Table 2.** The rate of change in PIPs in the intervention group versus the control group after adjusting for the GP subgroup characteristics. Estimates of IRRs showing the changes in PIPs per 100 prescriptions at post-intervention relative to 1 year pre-intervention obtained from the Poisson cluster effects regression model.

		
	**IRR (95% CI)**	***P*-value**
**PIPs at baseline (ref: control group)**		
Intervention	1.10 (0.97 to 1.25)	0.15
**Within-group reduction of PIPs relative to baseline**		
Control	0.93 (0.90 to 0.96)	<0.01
Intervention	0.80 (0.78 to 0.82)	<0.01
**^a^Reduction of PIPs after intervention (ref: control group)**		
Intervention	0.86 (0.83 to 0.90)	<0.01
Subgroups:		
**Age group, years (ref: 27–42 years)**		
43–48	0.85 (0.81 to 0.89)	<0.01
49–52	0.92 (0.87 to 0.97)	<0.01
53–56	1.00 (0.95 to 1.05)	0.94
57–68	1.21 (1.16 to 1.27)	<0.01
**Specialist (ref: non-specialist)**		
Specialist	1.04 (0.99 to 1.08)	0.13
**Type of practice (ref: single-handed)**		
Group practice	1.04 (0.99 to 1.08)	0.07
**Sex (ref: female)**		
Male	1.40 (1.35 to 1.44)	<0.01
**Practice setting (ref: rural)**		
Urban	1.15 (1.11 to 1.19)	<0.01
**Mean PIPs p 100 prescriptions at baseline (ref:** ≤1.6)		
1.7–2.0	1.40 (1.34 to 1.45)	<0.01
2.1–2.3	1.77 (1.71 to 1.84)	<0.01
2.4–2.9	2.10 (2.02 to 2.18)	<0.01
>2.9	2.40 (2.30 to 2.50)	<0.01
**Prescriptions per patient at baseline (ref: <8.7)**		
8.7–10.7	1.44 (1.38 to 1.51)	<0.01
10.8–12.8	1.96 (1.88 to 2.05)	<0.01
12.9–14.9	2.31 (2.20 to 2.42)	<0.01
≥15	2.97 (2.83 to 3.12)	<0.01

IRR = incidence rate ratio. PIP = potentially inappropriate prescription. Ref = reference. ^a^PIPs were 14% (1.0 to 0.86) lower in the intervention arm compared to the control at after intervention.

### Sub-group analyses of intervention effect

Table 3 shows that improvements in prescribing were highest among GPs aged 57–68 years, who were specialists, who worked in single-handed practices, who had 2.4 to 2.9 PIPs per 100 prescription at baseline, and among GPs with ≥15 prescriptions per older patient at baseline.

**Table 3.** Subgroup analyses comparing changes in prescription rates of PIPs by the seven GP-related characteristics. Unadjusted IRRs and their 95% CIs obtained from the Poisson regression model with cluster effects at CME group level showing the changes in PIPs within the subgroups, α = 0.007.

**Subgroups**	**Intervention group**		**Control group**	
	IRR (95% CI)	*P*-value	IRR (95% CI)	*P*-value
**Age group, years**				
27–42	0.92 (0.85 to 0.99)	0.043	1.12 (1.04 to 1.21)	0.004
43–48	0.78 (0.72 to 0.82)	<0.007	0.89 (0.84 to 0.95)	0.001
49–52	0.81 (0.77 to 0.86)	<0.007	0.83 (0.77 to 0.90)	<0.007
53–56	0.81 (0.83 to 0.85)	<0.007	0.92 (0.86 to 0.98)	0.006
57–68	0.77 (0.73 to 0.81)	<0.007	0.92 (0.87 to 0.97)	0.003
**Specialist**				
Non-specialist	0.84 (0.77 to 0.93)	<0.007	1.04 (0.96 to 1.13)	0.329
Specialist	0.80 (0.78 to 0.82)	<0.007	0.91 (0.88 to 0.94)	<0.007
**Type of practice**				
Single-handed	0.75 (0.68 to 0.83)	<0.007	0.91 (0.83 to 0.99)	0.034
Group	0.80 (0.78 to 0.83)	<0.007	0.93 (0.90 to 0.96)	<0.007
**Sex**				
Female	0.85 (0.80 to 0.90)	<0.007	0.93 (0.88 to 0.99)	0.027
Male	0.79 (0.77 to 0.81)	<0.007	0.92 (0.89 to 0.95)	<0.007
**Practice setting**				
Rural	0.80 (0.77 to 0.83)	<0.007	0.92 (0.88 to 0.96)	<0.007
Urban	0.80 (0.78 to 0.83)	<0.007	0.93 (0.90 to 0.97)	<0.007
**Mean PIPs per 100 prescriptions 1 year pre-intervention**				
≤1.6	0.88 (0.81 to 0.95)	0.001	1.09 (1.00 to 1.18)	0.041
1.7–2.0	0.83 (0.78 to 0.88)	<0.007	1.04 (0.97 to 1.12)	0.265
2.1–2.3	0.81 (0.76 to 0.85)	<0.007	0.85 (0.80 to 0.90)	<0.007
2.4–2.9	0.74 (0.70 to 0.78)	<0.007	0.89 (0.84 to 0.93)	<0.007
>2.9	0.80 (0.76 to 0.83)	<0.007	0.89 (0.83 to 0.96)	0.004
**Prescriptions per patient in the 1 year pre-intervention period**				
<8.7	0.90 (0.83 to 0.98)	0.016	1.16 (1.06 to 1.27)	0.001
8.7–10.7	0.83 (0.77 to 0.88)	<0.007	0.87 (0.81 to 0.93)	<0.007
10.8–12.8	0.80 (0.76 to 0.84)	<0.007	0.95 (0.88 to 1.02)	0.190
12.9–14.9	0.80 (0.76 to 0.84)	<0.007	0.92 (0.87 to 0.97)	0.004
>14.9	0.77 (0.73 to 0.80)	<0.007	0.88 (0.83 to 0.93)	<0.007

IRR = incidence rate ratio.

When the interaction between the intervention and each sub-group was tested, the overall tests were significant (*P*<0.01); hence, the results of the subgroups with *α* * set at 0.007 are reported. Reductions of PIPs were observed in all GPs aged ≥43 years in both the control and the intervention group (*P*<0.007). However, a 12% significant increase of PIPs was observed among GPs aged 27–42 years in the control group (*P* = 0.004).

PIPs decreased by 20% among specialist GPs in the intervention group and 9% among specialist GPs in the control group. Although a significant reduction (16%; *P*<0.007) of PIPs was observed among non-specialist GPs in the intervention group, changes of PIPs among non-specialist GPs in the control group were not significant.

In the intervention arm, a 25% reduction of PIPs was observed among GPs in single-handed practices compared to a 20% reduction of PIPs among GPs in group practices (*P*<0.007). The results for GPs from single-handed practices in the control arm were not significant (*P* = 0.034), as shown in Table 3. The analysis also showed significant improvements among GPs in the intervention arm, regardless of the mean number of PIPs per 100 prescriptions at baseline. The largest improvement in this group was observed among GPs who had between 2.4 and 2.9 PIPs per 100 prescriptions at baseline. However, the findings in the control group were not consistent. For example, significant improvements were observed among GPs who had ≥2.1 PIPs per 100 prescriptions at baseline. Although not statistically significant, an increase of PIPs at post-intervention was observed among GPs with <2.1 PIPs per 100 prescriptions at baseline.

Significant reductions of PIPs were observed in the other subgroups in the intervention arm with the exception of GPs who had <8.7 prescriptions per older patient at baseline (see Table 3).

## Discussion

### Summary

This study investigated whether GP characteristics influenced rates of PIPs after an educational intervention. It was found that improvements in prescribing were highest among GPs aged 57–68 years, who were specialists, who worked in single-handed practices, who had 2.4 to 2.9 PIPs per 100 prescription at baseline, and among GPs with ≥15 prescriptions per older patient at baseline.

The educational intervention included an exposure of each GP’s prescribing profile in a CME group, which may have strengthened their motivation for change. Yet another explanation for a greater reduction in PIPs in patients with a high number of PIPs at baseline is that there were more changes to be made in these patients.

A possible explanation for the high reduction rate of PIPs among the oldest GPs could be that this group more commonly prescribes 'older' drugs, even if newer and possibly safer alternatives are available. Several of the drugs used to measure inappropriateness in the *Rx*-PAD study had been in the market for decades. The *Rx-*PAD study demonstrated that being a young doctor was associated with a lower proportion of PIPs at baseline, while their older colleagues were more likely to prescribe inappropriately.^8^ That GPs in certain age groups and GPs with a high rate of PIPs at baseline improved most due to the intervention may suggest that future interventions could possibly be more cost effective if they are tailored towards a subgroup of GPs instead of targeting all. In a study analysing factors associated with adolescents non-response to an asthma management programme, Joseph *et al* ^15^ argue in favour of subgroup analyses to identify differences in baseline characteristics associated with the primary outcome in a randomised trial.^10^ This may be particularly useful if such characteristics are used as basis for creating submodules within the trial through which additional, theory-based strategies could be administrated.^10^

### Strength and limitations

To the best of the authors' knowledge, this is the first GP-based intervention study to investigate the influence of GPs’ characteristics on their change of prescribing practice for older patients across a broad spectrum of therapeutic areas. The *Rx*-PAD study was a large-scale intervention including about one-tenth of all Norwegian GPs (there were around 4100 GPs in Norway at the time of the study). This strengthens the external validity of the current results. The full model results from the *Rx*-PAD study are robust, with high power and a cluster randomised design.

Since the GPs recruited in an RCT are not a homogeneous sample, it is not surprising that their responses to an educational intervention vary in many ways. Thus, it may be difficult to apply the results of an educational intervention to individual GPs without considering their individual characteristics. However, results from subgroup analyses are usually unreliable because of reduction in statistical power, which may lead to incorrect conclusions. By formally testing the interactions between the intervention and the subgroups, in addition to using a strict *P*-value of 0.007, this study managed to control for multiple testing, hence reducing false negatives and positives. That the GPs in the control group also improved their prescribing practice for older patients may partly represent a Hawthorne effect in response to the fact that they knew they were studied.^16^ Adding to this is the fact that the control group was allocated to another educational intervention with focus on prescribing quality, although within another therapeutic field (that is, antibiotic use for respiratory tract infections).^12^ This may possibly have had effects on their overall prescribing quality.

Despite the age of the data, the results of this study are still relevant; firstly, because the continuous need for improved prescription quality for older people is quite similar today as it was 10 years ago. Secondly, and most important, the focus in this study was to explore how that intervention influenced prescribing patterns among GPs, related to individual and practice setting characteristics.

### Comparison with existing literature

To the authors' knowledge, few if any studies with a comparable size and design have explored the intervention effects by variables related to the GPs (age, sex, and specialist status) and their practices (single-handed versus group, and urban versus rural), and baseline performance regarding the target of an intervention. The effects of audit and feedback on prescribing of drugs are reported to be small to moderate, and the relative effectiveness is likely to be greater when baseline adherence to recommended practice is low.^17^ This is consistent with this study's findings.

### Implications for research

In the control arm of the *Rx*-PAD study, inappropriate antibiotic prescribing practice strongly correlated with practice activity in terms of annual number of encounters.^12,18^ In addition to the explanatory variables used in the present report, it would therefore also have been interesting to correlate GPs’ prescribing patterns for older patients to their practice activity in terms of annual patient encounters. However, such data were not recorded for the present arm of the *Rx*-PAD study. For future research, the inclusion of explanatory variables that also may reflect practice activity more directly are recommended, as well as the amount of time spent per patient in the consultation room.
